# Antibody Responses to SARS-CoV-2 in Cancer Patients in New York City During the 2020 Wave of the COVID-19 Pandemic

**DOI:** 10.7759/cureus.102541

**Published:** 2026-01-29

**Authors:** Maria Gianniki, Yuxuan Li, William Glembocki, Marilia Bernardes, Yoginder Hinwar, Kaitlin Walsh, Genovefa Papanicolaou

**Affiliations:** 1 Department of Medicine, Infectious Diseases and Allergy Service, Memorial Sloan Kettering Cancer Center, New York City, USA; 2 Department of Pathology and Laboratory Medicine, Memorial Sloan Kettering Cancer Center, New York City, USA

**Keywords:** 2020-2021 pandemic, anti-sars-cov-2 antibodies, covid-19 in cancer patients, covid-19 pandemic, new york city, sars-cov-2 antibody response, sars-cov-2 natural infection

## Abstract

Introduction: New York City was the epicenter of the first wave of the COVID-19 pandemic, and cancer patients were among the most vulnerable groups. We characterized antibody response in a diverse cohort of cancer patients prior to the availability of COVID-19 therapeutics. We report: (1) antibody response to COVID-19 by cancer type; (2) trend of SARS-CoV-2 IgG spike antibodies over time in cancer patients with COVID-19 diagnosis during 2020-2021.

Methods: Single-center, prospective, observational study of patients treated for cancer at Memorial Sloan Kettering Cancer Center with symptomatic, laboratory-confirmed COVID-19. Serial blood specimens were collected after COVID-19 diagnosis and before administration of any COVID-19 vaccine or monoclonal antibody. IgG was determined using the AdviseDx SARS-CoV-2 IgG II assay. Antibody response was defined as a titer ≥50 AU/mL. Early antibody response was defined as <14 weeks, and late antibody response as >14 weeks after COVID-19 diagnosis. Group comparisons were performed using the Wilcoxon rank-sum test, while Fisher’s exact or chi-squared tests and ANOVA were used for categorical comparisons. A locally estimated scatterplot smoothing technique and a linear mixed-effects model were applied to assess the trend in IgG titers over time.

Results: Of 245 patients analyzed, 127 (51.9%) had solid tumors and 118 (48.1%) hematologic malignancies. Among patients with hematologic malignancies, 15 received B-cell-depleting therapies within 6 months of COVID-19 diagnosis. Overall, 181 (87%) patients had early antibody response, including 97 (92%) patients with solid tumors, 77 (88%) with hematologic malignancies without B-cell-depleting therapies, and 7 (50%) with hematologic malignancies who received B-cell-depleting therapies. The magnitude of early antibody response was significantly lower in patients who received B-cell-depleting therapies compared to both other groups. A total of 452 specimens were analyzed to evaluate the trend of IgG titers over time. A measurable decline is observed over time, with an estimated 14.437 AU/mL decrease per day post-COVID-19 diagnosis (p <0.001).

Conclusion: Overall, 87% of cancer patients had an early antibody response to COVID-19. Only 50% of patients with hematologic malignancies who received B-cell-depleting therapies developed an antibody response, and IgG titers were significantly lower in this group compared to both patients with solid tumors and patients with hematologic malignancies without B-cell-depleting therapies. We observed a progressive decline in IgG titers over time, with an estimated decrease of 14.437 AU/mL per day post-COVID-19 diagnosis. These findings offer valuable insights into antibody responses in cancer patients following COVID-19 infection.

## Introduction

The COVID-19 pandemic imposed unprecedented challenges on the medical and scientific communities and a heavy toll on hospitalizations and deaths. The lack of COVID-19 therapeutics, disruptions of supply chains, and healthcare delivery substantially intensified these challenges. During the 2020 wave of the COVID-19 pandemic, New York City was among the first and hardest-hit regions worldwide, with significant morbidity and mortality, especially among cancer patients [1,2].

Understanding the natural history of viral infections in people with impaired immunity has emerged as a critical area of investigation during the COVID-19 pandemic. The interplay between cancer and COVID-19 has raised critical concerns regarding susceptibility to infection, disease severity, and outcomes. While many studies have focused on viral load characteristics and persistence, data on the magnitude and duration of antibody response (abR) to natural infection among cancer patients remain limited [2].

We have conducted a single-center, prospective, observational study of patients treated for cancer at Memorial Sloan Kettering (MSK) Cancer Center with symptomatic, laboratory-confirmed (LC) COVID-19 between 2020 and 2021. The primary objective of this study was to characterize the frequency and magnitude of antibody responses following natural COVID-19 infection in cancer patients prior to the availability of COVID-19 vaccines and therapeutics. Secondary objectives were to compare antibody responses across cancer subtypes, as well as to assess longitudinal trends in IgG titers over time, using serial measurements obtained up to 52 ± 4 weeks post-infection.

## Materials and methods

Study design

This is a single-center observational study. Patients with cancer presenting with ≥1 respiratory symptom who tested positive in nasopharyngeal swab for SARS-CoV-2 by real-time polymerase chain reaction (RT-PCR) from March 2020 through March 2021 at Memorial Sloan Kettering Cancer Center (MSK) were included in the study. SARS-CoV-2 testing for patients presenting with one or more respiratory symptoms was at the discretion of the treating physicians as deemed appropriate for clinical care. There was no standardized symptom checklist. For patients with multiple positive tests, the date of COVID-19 diagnosis is the date of the first positive test.

Specimen Collection

The study was conducted under a biospecimen protocol that enables the use of banked specimens and prospective collections. The protocol was approved on 9/21/2021. Patients with COVID-19 were identified from the clinical microbiology database and enrolled in the study if they had banked specimens before 9/21/2021. For time-points after 9/21/2021, specimens were collected prospectively.

Planned time-points for measurement of antibody response were 2-6, 14 ± 2, 24 ± 4, 38 ± 4, and 52 ± 4 weeks after COVID-19 diagnosis and quarterly thereafter. Deviations from allowed windows were permitted due to the availability of banked specimens and schedule disruption of in-person visits during the pandemic. Plasma specimens were stored at -80°C and tested in batches. Specimens collected after administration of any COVID-19 vaccine or COVID-19 monoclonal antibodies were excluded from the analyses. The study was reviewed and approved by the MSK Institutional Review Board.

Laboratory methods

COVID-19 Diagnosis

COVID-19 diagnosis was laboratory-confirmed (LC) by a real-time reverse transcription polymerase chain reaction (PCR) performed at the Clinical Microbiology Laboratory at MSK. Briefly, nasopharyngeal swab samples were collected using flocked swabs (Copan Diagnostics, Murrieta, CA) and placed in viral transport media. SARS-CoV-2 RNA was detected using the Centers for Disease Control and Prevention (CDC) protocol targeting two regions of the nucleocapsid gene (N1 and N2), with the following modifications: Nucleic acids were extracted from specimens using the NUCLISENS EasyMag (bioMérieux, Durham, NC) following an off-board, pre-lysis step [3,4]. Real-time reverse transcription PCR was performed on the ABI 7500 Fast (Applied Biosystems, Foster City, CA) in a final reaction volume of 20-μL, including 5 μL of extracted nucleic acids. Samples were reported as positive if both the N1 and N2 targets were detected.

SARS-CoV-2 IgG Antibody Testing

Antibody testing was performed at the Department of Laboratory Medicine at MSK. IgG against trimeric spike protein (S) was measured using the AdviseDx SARS-CoV-2 IgG II assay on the Architect-i2000-analyzer (Abbott Laboratories, Abbott Park, IL). The assay has a sensitivity of 92% and a specificity of >99%. A SARS-CoV-2 IgG value ≥50 AU/mL was considered positive. The linear range of quantification was 50-25,000 AU/mL.

Definitions

Patients were categorized by cancer type into the solid tumor group (ST) and the hematologic malignancies group (HM). HM were further categorized by receipt of B-cell depleting therapy (rituximab or/and obinutuzumab) (BCD) <6 months prior to COVID-19 diagnosis, as HM w/BCD and HM w/o BCD for patients who received or did not receive BCD, respectively. 

Antibody response (abR) was defined as a SARS-CoV-2 IgG spike titer above the assay cutoff (≥50 AU/mL). Early abR was defined as abR ≤14 weeks from COVID-19 diagnosis, and late abR as abR >14 weeks after COVID-19 diagnosis. The result of the first specimen collected within 14 weeks of the COVID-19 diagnosis was used to define a positive or negative immune response. Four time frames from COVID-19 diagnosis to specimen collection were examined: <6, 6-14, 14-24, and >24 weeks. 

Statistical analysis

Descriptive statistics were reported as frequency for categorical variables and median/interquartile range (IQR) for continuous variables. Antibody response (abR) was analyzed as: (1) categorical variable (+/-), (2) continuous variable (abR magnitude expressed as IgG titer (AU/mL)), and (3) trend over time for the study period. The Fisher’s exact or Chi-squared tests were used for categorical comparisons. Group comparisons of the magnitude of abR were performed using the Wilcoxon rank-sum test. Kruskal-Wallis test was performed to compare IgG titers across the three groups (ST, HM w/BCD, HM w/o BCD). Statistical significance was defined as p < 0.05. To visualize the trend of IgG titers over time during the study period, a locally estimated scatterplot smoothing (LOESS) technique was applied. The smoothing function was fitted to all available data points, treating each observation independently without incorporating patient-level random effects. A linear mixed-effects model (LMM) was applied to assess the trend of changes in IgG titers following natural COVID-19 infection while accounting for repeated measurements for the same patient. This statistical approach accommodates unbalanced and irregularly timed measurements, allowing inclusion of participants with incomplete follow-up.

## Results

Patients and specimens

The study cohort consists of 245 patients with LC COVID-19 between March 2020 and March 2021. Table 1 shows the baseline characteristics of the study cohort. 

**Table 1:** Baseline characteristics of the cohort (n = 245 patients).

Characteristic	N (%)
Age, median years (range)	60.3 (19–89)
Gender, male	124 (50.6%)
Race	
White	171 (69.8%)
Black or African American	25 (10.2%)
Asian	16 (6.5%)
Unknown/other	33 (13.5%)
Cancer type	
Hematologic malignancies (HM)	118 (48.2%)
B-cell depleting therapy (w/BCD)	15 (12.7%)
No B-cell depleting therapy (w/o BCD)	103 (87.3%)
Solid tumors (ST)	127 (51.8%)
First specimen collected <14 weeks of COVID-19 diagnosis (early)	208 (84.9%)

Of 245 patients, 127 (51.2%) had ST and 118 (50.8%) had HM. Fifteen (12.8%) patients with HM had received BCD (w/BCD). A total of 452 specimens were analyzed. The median number of specimens per patient was 2 (range: 1-4). The first specimen was collected a median of 7.8 weeks post-COVID-19 diagnosis (IQR: 5-11.6, range: 1.9-49.7).

Analyses of early antibody response (abR)

**Figure 1:**
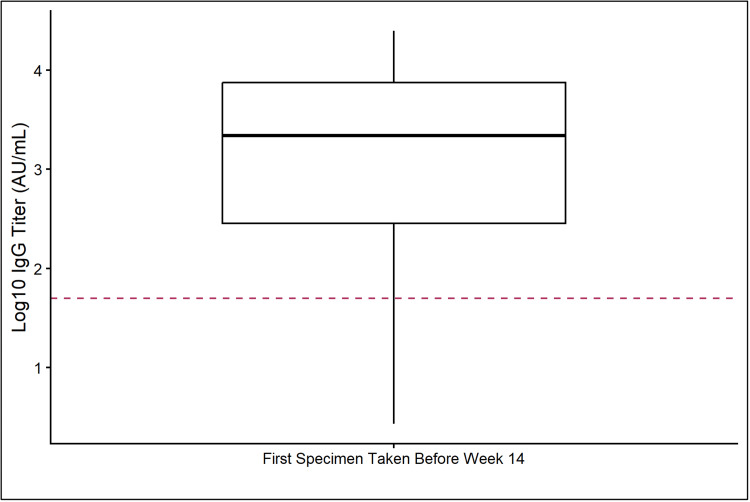
Magnitude of antibody response (abR) (IgG log10 AU/mL) by week 14 after COVID-19 diagnosis (total n=208 patients). Two hundred and eight patients had an abR available by week 14 after COVID-19 diagnosis. The boxed area contains the 25th and 75th quartiles. The solid horizontal line represents the median (2187 AU/mL log10 transformed to 3.34). The dotted line represents the cutoff (≤50 AU/mL).

Of 208 patients with ≥1 early specimen available, 181 (87%) had abR, including 97 (92%) of ST, 7 (50%) of HM w/BCD, and 77 (88%) HM w/o BCD. The median IgG titer was 2187 Au/mL, interquartile range (IQR) (279-7475) (Figure 1).

**Figure 2:**
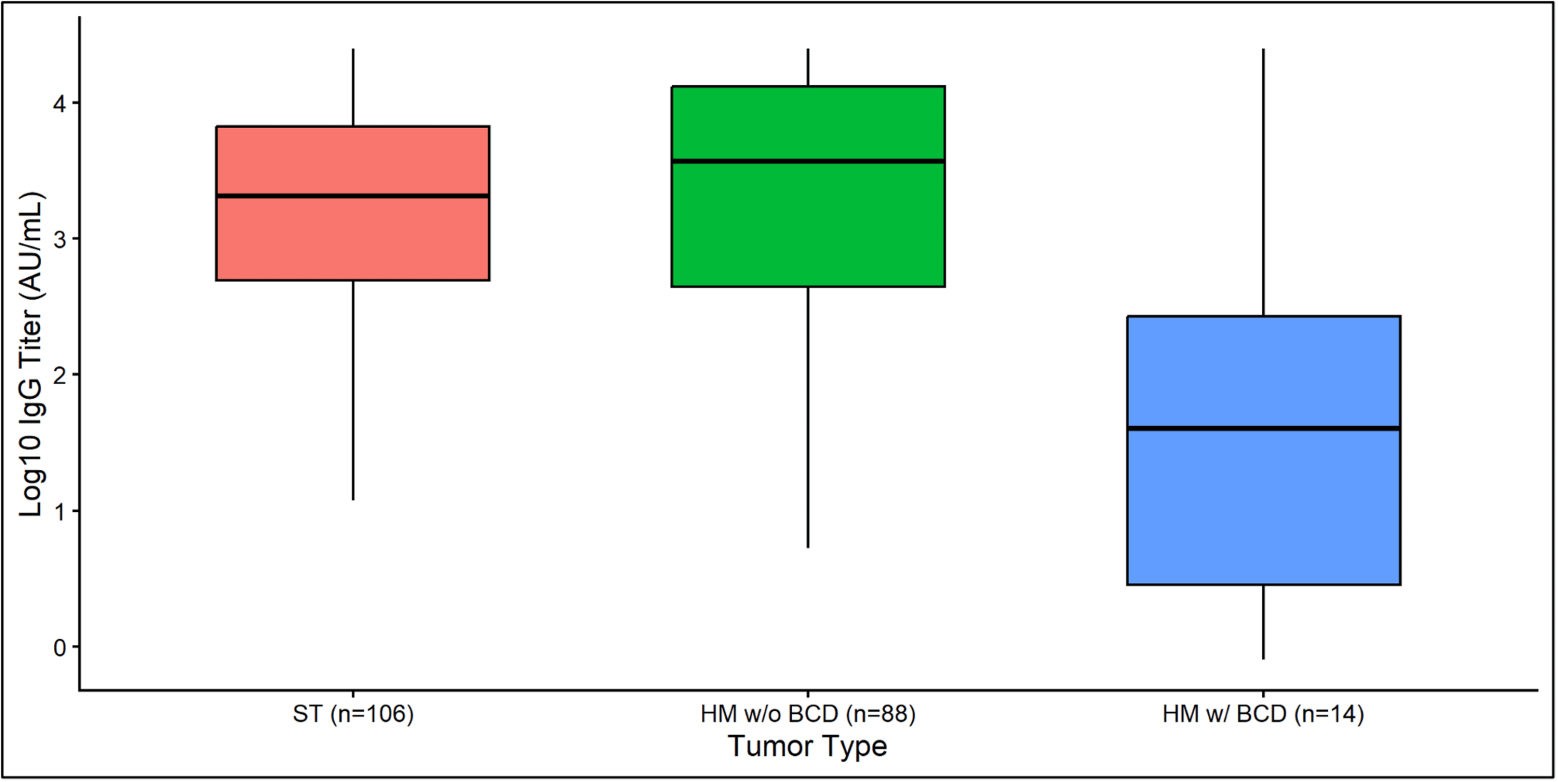
Comparison of early IgG titer (AU/mL) by cancer type. Early was defined as <14 weeks from COVID-19 diagnosis. When more than one specimen was available within this frame, the earliest specimen was included in the analysis. Log10 values of the raw titer medians and quartiles were used for visualization. Pairwise comparisons of early IgG titers were performed using the Wilcoxon rank-sum test with continuity correction, and p-values adjusted for multiple comparisons using the Bonferroni method.

We compared the IgG titers across the three patient groups (Figure 2). The median (IQR) for ST was 2050 AU/mL (495-6658), for HM w/o BCD 3625 (430-12,861), and for HM w/BCD 41 AU/mL (3-269). By pairwise comparison, HM w/BCD had lower early IgG titers compared to both ST (Wilcoxon rank-sum test: W = 1151.5, p = 0.0025) and HM w/o BCD (W = 3983.5, p = 0.0020). 

Trend of antibody response (abR) over time

**Figure 3:**
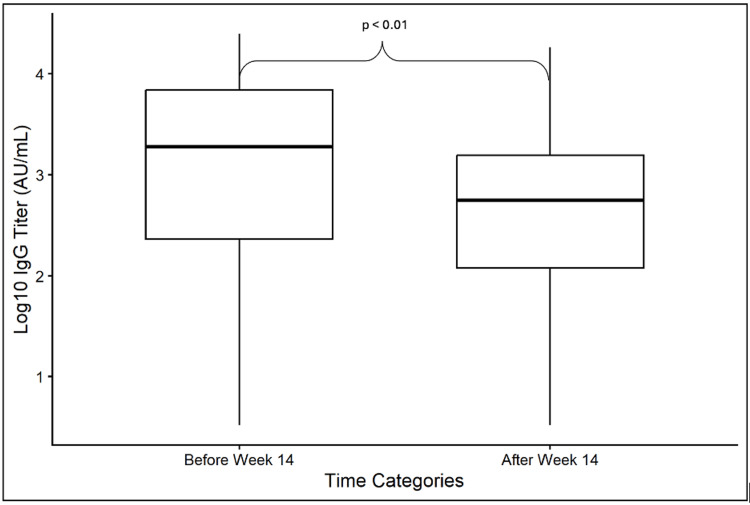
Comparison of IgG titer (log10 AU/mL) measured <14 weeks and >14 weeks from COVID-19 diagnosis among patients with paired specimens. For samples collected <14 weeks from COVID-19 diagnosis, the first available specimen was included; for samples collected >14 weeks, the last available specimen was included. Comparisons were performed using the Wilcoxon rank-sum test with continuity correction.

First, we compared IgG titers collected before and after 14 weeks from COVID-19 diagnosis, in 123 patients who had specimens collected at both timeframes. The median time to collection for early abR was 7.7 weeks (5.1-9.9) and for late abR (>14 weeks) 38.7 weeks (30-46.6). The late IgG titer was lower compared to the early IgG titer (Wilcoxon rank-sum test: W = 9722, p <0.01) (Figure 3).

**Figure 4:**
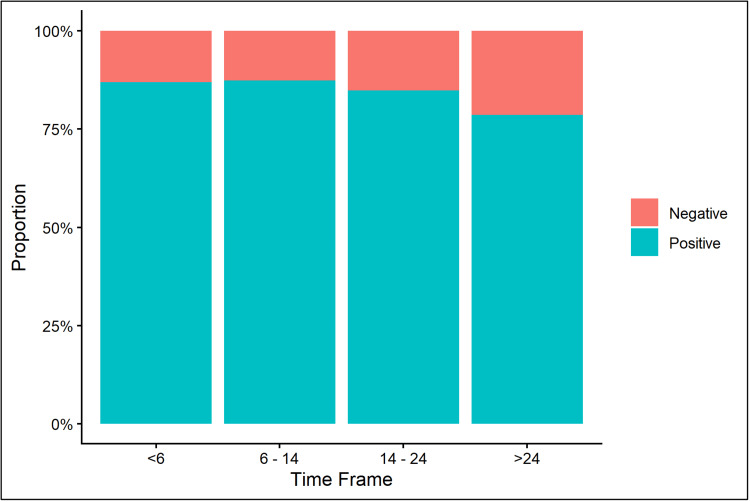
Proportion of positive antibody response (abR) specimens by timeframe (total n = 452 specimens).

Next, we analyzed the trend in IgG titer over time for all available specimens. A total of 452 specimens were included in these analyses. The median time to specimen collection was 15.8 weeks (IQR: 7.1-33) (range: 1.9-74.6 weeks) after COVID-19. Of the 452 specimens, 229 and 223 were from ST and HM patients, respectively. Four timeframes were examined (<6, 6-14, 14-24, and >24 weeks). Figure 4 shows the proportion of abR specimens by timeframe. 

The proportions of abR and IgG titers by cancer type and timeframe are summarized in Table 2.

**Table 2:** Proportion of abR and IgG titer (AU/mL) by cancer type and timeframe from COVID-19 diagnosis. Each row indicates a timeframe (in weeks) from the COVID-19 diagnosis. IgG titers are presented as median (IQR), and abR is expressed as percentages. Comparisons of IgG titers were performed using the Wilcoxon rank-sum or Kruskal-Wallis test, as appropriate. Comparisons of abR proportion were performed using the chi-square or Fisher's exact test, as appropriate. abR: antibody response positive; IQR: interquartile range.

2a. Solid tumors (n = 127 patients; 229 specimens)				
Timeframe (weeks)	Number of specimens	abR n (%)	IgG titer (AU/mL)	
			Median (IQR)	Range
<6	31	25 (80.65)	2258 (979-3999)	84.4-25,000
6–14	79	76 (96.20)	2230 (790–7117)	58–25,000
14–24	32	27 (84.37)	1154 (565–2837)	143–25,000
> 24	87	72 (82.76)	683.45 (305–1511)	50–14,119
2b. Hematologic malignancies (n = 118 patients; 223 specimens)				
Timeframe (weeks)	Number of specimens	abR n (%)	IgG titer (AU/mL)	
			Median (IQR)	Range
<6	53	48 (90.57)	4947 (988–17,997)	56–25,000
6–14	55	41 (75.55)	3767 (1357-12,152)	51–25,000
14–24	34	29 (83.29)	2147 (881–4076)	78–18,276
>24	81	60 (74.07)	1004 (300–1625)	50–6440

Due to the limited number of specimens from HM w/BCD per timeframe, HM w/BCD and w/o BCD are reported under HM. For ST, abR ranged from 80.65% to 96.2% across all timeframes. For HM, the highest percentage of abR was observed in the timeframe of <6 weeks (90.57%) and the lowest at >24 weeks (74.07%). abR and IgG titers did not differ significantly across cancer types or timeframes (all p-values >0.05).

**Figure 5:**
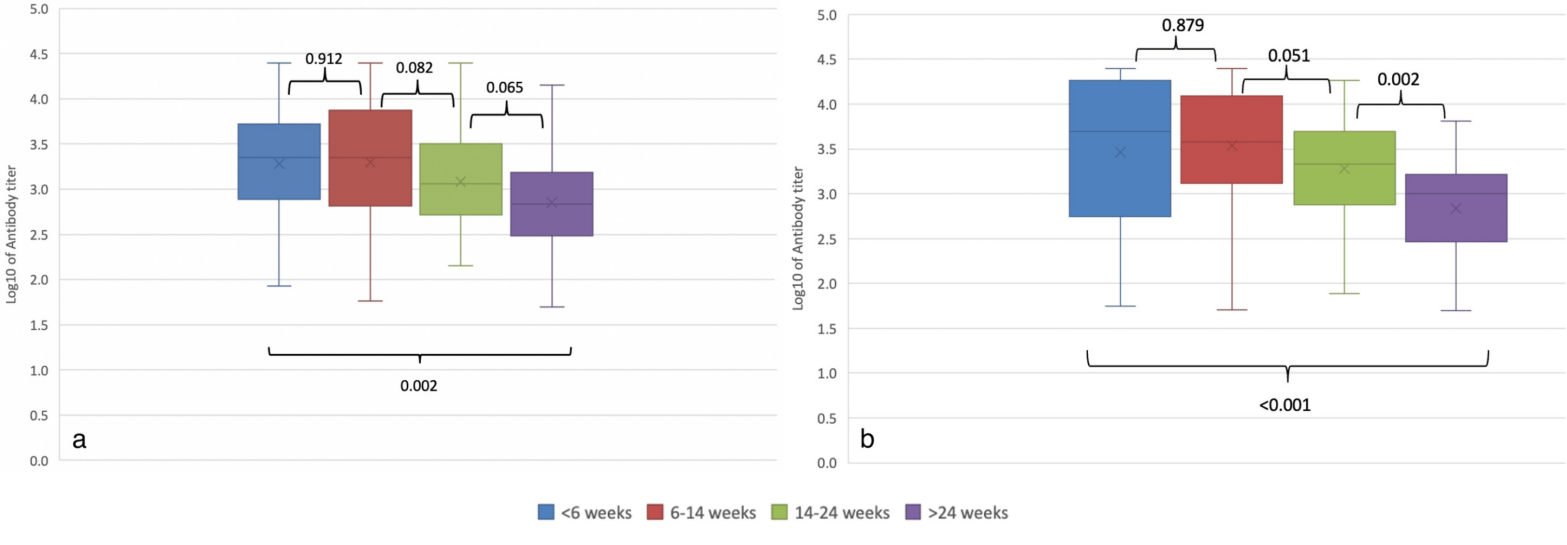
Comparison of IgG titer (log10 AU/mL) by timeframe of collection. (a) Solid tumors (total 200 abR specimens), (b) hematologic malignancies (total 178 abR specimens). Pairwise comparison was performed between contiguous timeframes and between the first and last timeframes, using the Wilcoxon rank-sum test with continuity correction. P-values are shown. Each boxplot extends from the 25th to the 75th percentile of each time interval group’s distribution of values (IQR). The horizontal lines inside each box denote the median. The whiskers extend to the furthest measured IgG titers data point of each time interval group within 1.5 × IQR. abR: antibody response positive.

Figure 5 shows the comparison of IgG titers across timeframes by cancer type. For ST, there was a decrease in IgG titer at >24 weeks compared to prior timeframes (Wilcoxon rank-sum test: W = 1277, p = 0.002). For HM, a stepwise decline was observed after 14 weeks; from 6-14 weeks to 14-24 weeks (W = 759, p = 0.05) and from 14-24 weeks to >24 weeks (W = 1227, p = 0.002). 

**Figure 6:**
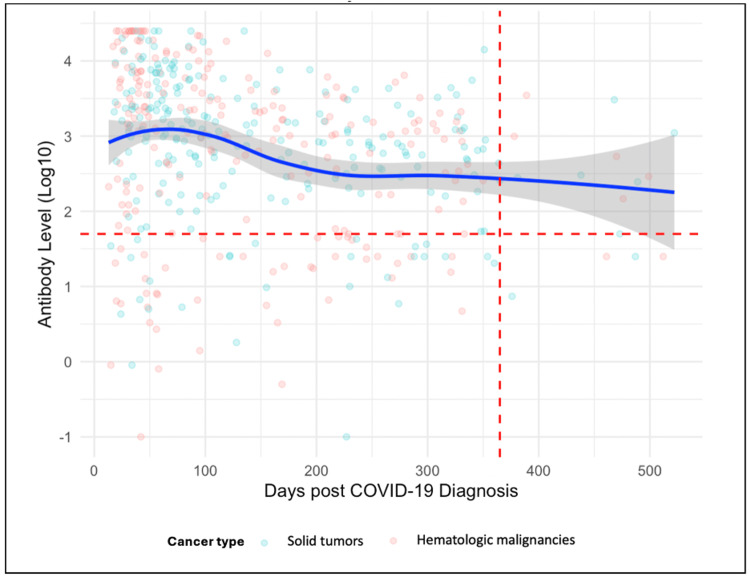
Smoothed trend of IgG titer (log10 AU/mL) from COVID-19 diagnosis (total, n = 452 specimens). A locally estimated scatterplot smoothing (LOESS) technique was applied. This smoothing trend (blue line) highlights the overall pattern of antibody decline, with a 95% confidence interval (gray shading). The red vertical dashed line represents the 365 days post-COVID-19 diagnosis, while the red horizontal dashed line represents the 50 AU/mL cut-off (log10).

Figure 6 visualizes the decline in IgG titers over time, after applying a locally estimated scatterplot smoothing (LOESS) technique. A measurable decline in IgG titers over time is observed, with a 14.437 AU/mL decrease per day post-COVID-19 diagnosis (p <0.001). 

## Discussion

Because of the availability of SARS-CoV-2 PCR at Memorial Sloan Kettering Cancer Center since early March 2020, we were one of the first institutions to report on laboratory-confirmed cases of COVID-19 in cancer patients in New York City (NYC) [2]. We previously reported the clinical determinants of severity and COVID-19-associated mortality in our patient population. Data on the magnitude and duration of antibody responses after COVID-19 prior to vaccination remain limited in cancer patients [2]. The present study reports the proportion of patients with positive antibody response and IgG titer trend over time in 245 cancer patients with a confirmed COVID-19 diagnosis between 2020 and 2021 treated at our Institution.

Overall, 87% of the patients had early abR, including 92% ST, 88% HM w/o BCD, and 50% of HM w/BCD. The magnitude of early abR in HM w/BCD was significantly lower compared to both ST and HM w/o BCD. We observed a significant decline in IgG titers over time, with a 14.437 AU/mL decrease per day post-COVID-19 diagnosis.

Our study makes several clinically relevant observations. First, despite concerns about impaired immunity, 92% of ST and 82% of HM patients had abR in the first 14 weeks after COVID-19 diagnosis. This finding suggests that even in the context of cancer and associated treatments, many patients were able to mount a positive abR to SARS-CoV-2. The same findings were initially presented in early pandemic studies; a study of MSK in December 2020 showed that 66% of hematopoietic cell transplant patients developed antibodies, while a similar study of multiple myeloma patients at Mount Sinai Health System showed that the majority of these patients mounted an antibody response to SARS-CoV-2 [3,4].

Published data on COVID-19 seropositivity in cancer patients vary widely. Rates as low as 1.6%-3.6% have been reported [5,6]. At the same time, a study in Spain concluded that 27.1% of patients receiving chemotherapy and 38.5% of patients with other treatments (hormone therapy, immunotherapy, and targeted treatment) developed antibodies, while a study in China found that the prevalence of IgG was 72.5% in cancer patients with COVID-19 [7,8]. Considerable variation in seropositivity had already been observed in the results of the early studies during the first pandemic wave [9]. This variation could be attributed to different cancer types, treatment regimens, and timing of infection relative to therapy, as well as to demographic, geographic, and temporal factors. Test accuracy, sensitivity in reliably detecting antibody responses to mild infections, the choice of antibody, and target antigen selection could also explain the wide variability of true seropositivity in some seroepidemiological studies. In addition, evidence published during the first wave of the pandemic described a clear correlation between the severity of SARS-CoV-2 disease and the magnitude of the immune response [10,11].

A unique aspect of our study is that it was conducted in New York City from March 2020 to March 2021. The unique demographics and high incidence of COVID-19 during the study period may have impacted the magnitude and persistence of the antibody response landscape. Higher exposure risk, varying viral loads, and coexisting health disparities and possibly reinfections may have influenced infection severity and immune response kinetics in cancer patients.

Patients treated with BCD had a lower magnitude of early antibody response, in agreement with previously published studies on COVID-19 vaccine responses in the setting of BCD [12,13]. We show that BCD impacts the abR and abR magnitude after natural COVID-19 infection. In addition to B-cell depleting agents, studies have suggested that tumor necrosis factor (TNF) inhibitors and methotrexate also attenuate SARS-CoV-2 antibody production [13,14].

Our study confirms that the abR to natural infection SARS-CoV-2 is waning over time, consistent with Langerbein's and Hallek's study, which showed that patients with hematologic malignancies show short-lasting protection after SARS-CoV-2 infection with detection of specific antibodies for less than four months and signs of T-cell exhaustion, indicating a lack of long-term immunity and an increased risk of reinfection [15]. In addition, our study showed that in HM, the magnitude of abR >24 weeks post-COVID-19 diagnosis was significantly lower than at earlier timeframes. Our results are in agreement with those reported by Borgogna et al. in 2022 [16].

This study has limitations. Conducting the study during the early phase of the COVID-19 pandemic, we faced several substantial logistical challenges, including disruptions in routine cancer care, reduced frequency of in-person visits to the center, and staff shortages, which affected specimen collection, processing, storage, and retrieval. These factors account for the variability in the number and timing of specimens per patient, and batch testing was also occasionally delayed due to competing priorities of laboratory medicine during the pandemic. The single-center, observational design may limit generalizability, and inclusion of only symptomatic patients could introduce selection bias, as antibody kinetics in subclinical infections may differ from those in symptomatic disease. A potential bias, inherent to the longitudinal nature of our study, is that patients who died early after COVID-19 may be underrepresented. Consistent with real-world studies, the cohort was heterogeneous in terms of cancer diagnosis, stage, type of treatment, and corticosteroid use, and residual confounding from these factors cannot be fully excluded. Immune assessment was limited to measurement of SARS-CoV-2 IgG antibodies; neutralizing antibody activity and cellular immunity were not evaluated, which may restrict interpretation of protective immunity and immune durability. In addition, no universally established immunoglobulin G threshold exists to define protective immunity. Social determinants of health, such as distance from the treatment center or access to transportation, may also have influenced our results. Finally, as the study was conducted during the early “wild-type” SARS-CoV-2 era, the observed antibody responses may not fully generalize to infections caused by later variants, and the absence of a non-cancer comparator group limits the ability to place our findings in a broader comparative context.

Acknowledging these limitations, our study has several strengths. We studied a diverse cancer population after natural infection with the original SARS-CoV-2 L strain before any mutations, herd immunity, or availability of COVID-19 therapeutics (antivirals, vaccines, or antibodies). We conducted separate analyses by tumor type, highlighting potential differences in the magnitude and duration of immunity across ST and HM patients. Our findings of waning immunity after natural infection mirror the waning of immunity after vaccination and highlight the need for vaccination after infection or boosters in vaccinated patients at risk for severe COVID-19.

Specifically, the decline in immune response to SARS-CoV-2, particularly following natural infection or initial vaccination, has been a critical factor guiding the development and administration of COVID-19 vaccines. This diminishing immunity has underscored the importance of vaccine boosters in reinforcing and prolonging protective immunity [17-19]. Additional research is needed to define the relevant cutoff of protective immunity against COVID-19 after natural infection and vaccination. 

## Conclusions

In this single-center observational study of cancer patients with laboratory-confirmed SARS-CoV-2 infection prior to the availability of COVID-19 vaccines, the majority of patients with solid tumors and hematologic malignancies mounted an early antibody response following natural infection. However, patients receiving B-cell-depleting therapy demonstrated a significantly lower magnitude of antibody response. We demonstrate a progressive, significant decline in IgG titers over time, with an estimated decrease of 14.437 AU/mL per day post-COVID-19 diagnosis.

These findings have important clinical implications for cancer patients, particularly those receiving immunosuppressive therapies. The observed waning of antibody responses following natural infection supports the need for vaccination and boosters even in previously infected individuals. Future studies are needed to define the relevant cutoff of protective immunity against COVID-19 after natural infection and vaccination. 
